# Use of antenatal services and delivery care among women in rural western Kenya: a community based survey

**DOI:** 10.1186/1742-4755-3-2

**Published:** 2006-04-06

**Authors:** Anna M van Eijk, Hanneke M Bles, Frank Odhiambo, John G Ayisi, Ilse E Blokland, Daniel H Rosen, Kubaje Adazu, Laurence Slutsker, Kim A Lindblade

**Affiliations:** 1 University of Amsterdam, the Netherlands; 2 Kenya Medical Research Institute, Centre for Vector Biology and Control Research, Kisumu, Kenya; 3 Global AIDS Program, Centers for Disease Control and Prevention, Atlanta, GA, USA; 4 Division of Parasitic Diseases, National Center for Infectious Diseases, Centers for Disease Control and Prevention, Atlanta, GA, USA

## Abstract

**Background:**

Improving maternal health is one of the UN Millennium Development Goals. We assessed provision and use of antenatal services and delivery care among women in rural Kenya to determine whether women were receiving appropriate care.

**Methods:**

Population-based cross-sectional survey among women who had recently delivered.

**Results:**

Of 635 participants, 90% visited the antenatal clinic (ANC) at least once during their last pregnancy (median number of visits 4). Most women (64%) first visited the ANC in the third trimester; a perceived lack of quality in the ANC was associated with a late first ANC visit (Odds ratio [OR] 1.5, 95% confidence interval [CI] 1.0–2.4). Women who did not visit an ANC were more likely to have < 8 years of education (adjusted OR [AOR] 3.0, 95% CI 1.5–6.0), and a low socio-economic status (SES) (AOR 2.8, 95% CI 1.5–5.3). The ANC provision of abdominal palpation, tetanus vaccination and weight measurement were high (>90%), but provision of other services was low, e.g. malaria prevention (21%), iron (53%) and folate (44%) supplementation, syphilis testing (19.4%) and health talks (14.4%). Eighty percent of women delivered outside a health facility; among these, traditional birth attendants assisted 42%, laypersons assisted 36%, while 22% received no assistance. Factors significantly associated with giving birth outside a health facility included: age ≥ 30 years, parity ≥ 5, low SES, < 8 years of education, and > 1 hour walking distance from the health facility. Women who delivered unassisted were more likely to be of parity ≥ 5 (AOR 5.7, 95% CI 2.8–11.6).

**Conclusion:**

In this rural area, usage of the ANC was high, but this opportunity to deliver important health services was not fully utilized. Use of professional delivery services was low, and almost 1 out of 5 women delivered unassisted. There is an urgent need to improve this dangerous situation.

## Background

In 2000, it was estimated that approximately 529 000 women died from complications related to pregnancy or delivery [1]. The majority of maternal deaths occur in developing countries. Causes of maternal deaths include complications of abortion, obstetric complications such as hemorrhage, dystocia, eclampsia, sepsis and infections such as tuberculosis and HIV-1 [2,3]. Attendance at antenatal clinics (ANCs) and receipt of professional delivery care have been associated with a reduction in maternal deaths [4,5]. The ANC system in developing countries has been adapted from developed countries without formal evaluations of the impact of interventions in developing country settings. [6]. More recently, studies have been conducted to identify procedures proven to improve reproductive health [7-9]. These evaluations demonstrated that in low risk pregnancies (pregnancies with no abnormal obstetric or medical history), a decrease from 12 ANC visits, as had been previously recommended, to a less-costly 4 visit-schedule did not result in an increase in adverse maternal and perinatal events [10-12]. As a consequence, the World Health Organization now recommends a 4-visit ANC schedule for low risk pregnancies [13]. Other interventions shown to be beneficial to mother and child include routine iron and folate supplementation in areas with a high prevalence of anemia, serologic screening for and treatment of syphilis, routine measurement of fundal height, malaria prevention, and tetanus immunization [9]. To fully benefit from these interventions, it is important that women begin attending ANC early in pregnancy.

In many developing countries, the majority of births occur without the help of a skilled assistant (defined as a midwife, nurse trained as midwife, or a doctor) at home or in other non-hospital settings [14]. Home deliveries in the absence of skilled professional attendants have been associated with adverse infant and maternal outcome [15,16]. However, home deliveries without a skilled attendant are chosen or occur for a variety of reasons, including long distances or difficult access to a birth facility, costs of services and perceived lack of quality of care in a health facility [17-19]. In an attempt to improve care during home deliveries and reduce maternal mortality, traditional birth attendants (TBAs) have been trained in modern delivery care, with varying reports of success [20-22]. Presence of a professional attendant at each birth can lead to a marked reduction in maternal mortality and morbidity [15,16]; professional health care during childbirth is one of the process indicators to assess progress towards the Millennium Development Goal of improving maternal health [23].

We conducted a community-based survey in rural western Kenya, where anemia, malaria and HIV rates are high, among a representative sample of women who had recently delivered, to identify the locations where they had sought care during their pregnancy and delivery and the range of services they had received. This information will help to improve maternal health care in this area.

## Methods

The survey was conducted in the rural area of Asembo (Rarieda Division) and Gem (Wagai and Yala Divisions) in western Kenya. Asembo has a population of approximately 55,000 people living in 79 villages. Gem has 75,000 inhabitants living in 144 villages. This area historically experienced intense perennial malaria transmission before the initiation of a large community-based trial of insecticide treated bed nets (ITNs) that reduced malaria transmission considerably [24,25]. Following this trial, a health and demographic surveillance system (HDSS) was established in the same area [26]. All births, deaths, pregnancies and in- and out migrations are recorded and updated every four months. Socio-economic information and educational level are obtained and updated annually. All households who participate in the HDSS receive ITNs; free retreatment is available in each village every 9 months.

Using a population-based sampling frame of women in the HDSS who had recently delivered, we selected a random sample of 730 women. We estimated that a sample size of 730 was required to calculate the proportion of those using antenatal or delivery care in both Asembo and Gem within 5 percentage points of the true proportion, assuming the true proportion was 70% and that 12% of women would not be available. Trained interviewers visited the selected women at home and administered a standardized questionnaire in the local language. Participants were asked if they had visited an ANC during their last pregnancy. If they answered "Yes," they were asked to specify their reasons for doing so; this question was repeated until no new reasons were reported. Answers were tick marked in predefined options or described under "other reasons." Interviewers were instructed not to probe with options. Socio-economic information was obtained from the HDSS. Information on household assets was used to derive a wealth index using the method developed by Filmer & Pritchet (2000) [27]. All households were categorized by quintile: a medium/low socio-economic status (SES) was defined as an SES in the bottom 3 quintiles of the wealth index.

Differences in proportions were compared using the Chi-square test or Fisher's exact test when appropriate, and differences in means were compared using the Student's *t*-test (SPSS for Windows 11.0, SPSS Inc. Chicago, Illinois). A two-sided *P*-value < 0.05 was considered statistically significant. Odds ratios with 95% confidence intervals were calculated. Results were stratified by parity, age, level of education (< 8 years versus ≥ 8 years of education) area of residence (Asembo versus Gem), and history of child death or previous stillbirth. Infrequent usage of ANC was defined as having made < 3 ANC visits, the minimum number needed to benefit from intermittent preventive treatment with sulfadoxine-pyrimethamine for malaria. Primiparae were women who had delivered for the first time, multiparae were women who had delivered more than once. Grande multiparae were defined as women who had delivered 5 or more times, given that a high number of previous deliveries is an obstetric risk factor. Logistic regression was used to assess the effect of various factors on ANC attendance, place of delivery and type of attendant at delivery. The effect of the following factors was examined: maternal age, parity, marital status, socio-economic status, education level, area of residence (Gem versus Asembo), distance to the ANC and history of stillbirth or child death. Factors significant in the univariate analysis were included in the multivariate analysis.

The project was reviewed and approved by the institutional review boards of the Centers for Disease Control and Prevention (Atlanta, GA) and the Kenya Medical Research Institute (Nairobi, Kenya). All women who participated gave written informed consent after reading through the consent form with the interviewer; participants who could not write indicated their consent by a fingerprint, which was witnessed by the interviewer.

## Results

### Study population

Interviews were conducted in December 2002. Of the 730 women who were selected, 8 were found not to have recently delivered and were excluded. Eighty-two (11.4%) of the remaining 722 eligible women could not be reached during the period of survey. An additional 3 women (0.4%) had died in the interval between delivery and interview and information from 2 women (0.3%) was assessed to be unreliable. The 87 women (median age 22 years) who could not be interviewed (12% of the eligible sample) were significantly younger than the 635 women included in the analysis (median age 25 years, *P *< 0.001, Mann-Whitney U test). The time interval between interview and last delivery was less than 6 months for 89% of the interviews and the maximum interval was 11 months. The number of years of education, socio-economic status, and time interval between interview and delivery were similar between women interviewed and those not interviewed (number of years of education 6.8 versus 6.4 years, respectively, *P *= 0.5; a low/medium SES 57% versus 57%, respectively, *P *= 1.0; interval between delivery and interview: 4.0 versus 4.2 months, respectively, *P *= 0.8, Mann-Whitney U test).

Of the 635 women enrolled, 98% were members of the Luo ethnic group. The mean age (standard deviation, SD) was 26.5 (6.9) years; 40 women (6%) were less than 18 years of age. The median number of pregnancies was 4 (range 1–15). Of the 621 singleton deliveries, 15 (2%) were stillbirths and by the time of the interview 27 live born infants (4%) were reported to have died.

### Care during pregnancy

A total of 571 women (90%) visited an ANC at least once; 34 different ANCs in or around the area of the survey were visited. The number of antenatal visits during pregnancy ranged from 1 to 10, with a median of 4. One or two visits only were made by 46 (8%) and 69 (12%) of the women respectively. Among the 559 ANC attendees who could recall when they first attended, 78 women (14%) started ANC visits in the first trimester, 355 women (64%) started in the second trimester, and 126 women (23%) in the third trimester.

Most (87%) women decided for themselves to visit the ANC; the husband, mother or mother-in-law suggested attending the ANC for only a few (5%, 4%, and 2% respectively). Young women and primiparae were more likely to have been advised to attend by their mothers or mothers-in-law than were older or multiparae women (e.g. among young women, 9% were advised to attend by her mother-in-law versus 1% among older women, OR 11.0, 95% CI 3.4–35.9). Many women (67%) gave more than 1 reason to visit an ANC; the reasons most frequently mentioned were: to check the position, condition or growth of the baby (83%); to detect maternal problems and to be treated when sick (55%); to get a tetanus injection (24%); and to get an ANC card (18%). Participants expressed the belief that medical staff in health facilities treat pregnant women better if they attend with an ANC card, particularly if the card shows evidence of multiple visits. Eleven percent of the women mentioned that they appreciated the health information the ANC provided in the form of talks or posters.

Most women (93%) reported walking to the ANC; walking times ranged from 1 minute to 3 hours (median: 40 minutes). Although distance was cited as a barrier to ANC use, 102 women (18%) did not visit the nearest ANC. Major reasons given for attending a more distant ANC included better perceived care (78%), or lower cost (13%). Seven percent of women visited more than one ANC, usually because of a temporary move or because services were thought to be better in the second ANC. In addition, 18% of women sought other sources of care during their pregnancies, such as TBAs (65%), religious leaders (14%), herbalists (13%), or traditional healers (8%).

Incomplete and inadequate services at the ANC visited were complaints mentioned by 29% of women who visited an ANC. These women were more likely to start ANC in the third trimester (28% versus 20% among women who did not complain about the quality of ANC services, OR 1.5, 95% CI 1.0–2.3, *P *= 0.05), and had a lower median number of ANC visits (3), although this was not statistically significant (Mann-Whitney U test, *P *= 0.1).

A total of 64 women (10%) never attended an ANC during their most recent pregnancy. The most frequently mentioned reasons for not attending were not seeing the need to attend (36%), expenses of transport or the cost of the ANC (27%), belief that the care was not adequate (22%), and distance to the ANC (14%). Of the 64 who did not attend, 27% sought alternative care during pregnancy from sources such as TBAs, religious persons, or herbalists.

Factors associated with not attending an ANC are summarized in table 1. Adolescents (women < 18 years) and older women (> 34 years) were the least likely to attend. In a multivariate model, only < 8 years of education (adjusted odds ratio [AOR] 3.0, 95% CI 1.5–6.0) and medium/low SES (AOR 2.8, 95% CI 1.5–5.3) remained associated with never attending an ANC.

**Table 1:** Association between maternal characteristics and not visiting an ANC, Asembo/Gem, Western Kenya, December 2002

	n (%*) who visited an ANC (N = 571)	N (%*) who did not visit an ANC (N = 64)	Factors associated with not visiting an ANC, univariate analysis OR (95% CI)	Factors associated with not visiting an ANC, multivariable analysis AOR (95% CI)
Age				
< 18 years	34 (6.0)	6 (9.4)	1.72 (0.67–4.43)	1.55 (0.36–6.72)
18–19 years	79 (13.8)	1 (1.6)	**0.12 **(0.02–0.92)	0.16 (0.02–1.27)
20–29 years	282 (49.4)	29 (45.3)	Reference	Reference
30–34 years	99 (17.3)	9 (14.1)	0.88 (0.40–1.93)	0.53 (0.21–1.32)
> 34 years	77 (13.5)	19 (29.7)	**2.40 **(1.28–4.51)	1.16 (0.51–2.67)
Parity				
Para 1	103 (18.0)	8 (12.5)	1.20 (0.49–2.91)	1.25 (0.33–4.80)
Para 2–4	231 (40.5)	15 (23.4)	Reference	Reference
Para ≥ 5	237 (41.5)	41 (64.1)	**2.66 **(1.44–4.95)	1.95 (0.85–4.49)
Marital status				
Single/widow	79 (13.9)	9 (14.1)	1.02 (0.48–2.14)	
Married	491 (86.1)	55 (85.9)	Reference	
Socio-economic status				
Low/medium	303 (54.5)	49 (76.6)	**2.73 **(1.49–4.98)	**2.82 **(1.49–5.34)
High	253 (45.5)	15 (23.4)	Reference	Reference
Education level				
< 8 years	291 (52.6)	51 (81.0)	**3.83 **(2.00–7.33)	**3.02 **(1.51–6.04)
≥ 8 years	262 (47.4)	12 (19.0)	Reference	Reference
Residence				
Asembo	293 (51.3)	26 (40.6)	0.65 (0.38–1.10)	
Gem	278 (48.7)	38 (59.4)	Reference	
Distance to ANC				
Walking < 1 hr	298 (52.2)	27 (42.2)	Reference	Reference
Walking 1 hr	128 (22.94)	16 (25.0)	1.38 (0.72–2.65)	1.13 (0.57–2.23)
Walking > 1 hr	107 (18.7)	20 (31.3)	**2.06 **(1.11–3.83)	1.55 (0.80–3.03)
Used bus or bike	38 (6.7)	1 (1.6)	0.29 (0.04–2.20)	0.40 (0.05–3.12)
Previous child-death				
≥ 3 times	38 (6.7)	9 (14.1)	**2.79 **(1.23–6.31)	1.40 (0.52–3.74)
1 or 2 times	180 (31.5)	25 (39.1)	1.63 (0.93–2.86)	1.14 (0.59–2.17)
None	353 (61.8)	30 (46.9)	Reference	Reference
Previous stillbirth				
Yes	65 (11.4)	8 (12.5)	1.11 (0.51–2.44)	
No	506 (88.6)	56 (87.5)	Reference	

Abbreviations: OR, odds ratio; AOR, adjusted odds ratio; CI, confidence interval; hr:hour. Significant (adjusted) odds ratios printed in bold.

*Column percentages

Factors associated with infrequent visits (< 3 times) among women who visited the ANC were similar to factors associated with not visiting at all, with the addition that being a single woman (single, separated, or divorced: AOR 2.5, 95% CI 1.3–5.0) was associated with making <3 visits to the ANC.

### Services offered by the ANC

Almost all women were examined and received a tetanus vaccination during their ANC-visits (Table 2). However, other preventive treatments, laboratory tests, and health education were not common, ranging from 67% for a blood pressure measurement to 3% for treatment for helminthiasis. One out of 5 women received malaria prevention (21%) or underwent a syphilis test (19%), and approximately half of the women received iron (53%) and folate (44%) supplementation. Among the 80 (14%) women who received health talks during ANC visits, the two most frequent topics were care during pregnancy (recommended diet, avoiding a heavy workload, importance of regular ANC attendance and a hospital delivery), and care for the newborn (e.g. diet, breastfeeding). Family planning (mentioned by 5 women), the use of bed nets to prevent malaria (3), and HIV prevention (3) were infrequent health education topics.

**Table 2:** Services offered in the ANCs visited during the last pregnancy, Asembo/Gem, Western Kenya, December 2002*

Services offered in ANC	n (%)
Palpation of the abdomen	562 (98.4)
Tetanus vaccination	555 (97.2)
Weight measurement	518 (90.7)
Blood pressure measurement	385 (67.4)
Iron supplementation	303 (53.1)
Hemoglobin measurement	281 (49.2)
Folic acid supplementation	253 (44.3)
SP (≥ 1 dose)	121 (21.2)
Syphilis test	111 (19.4)
Urine test	89 (15.6)
Information on danger signs during pregnancy	82 (14.4)
Health talk	80 (14.0)
Stool test	72 (12.6)
Antihelmintics	15 (2.6)

Abbreviations: ANC: antenatal clinic; SP: sulfadoxine-pyrimethamine

*Note: no information was obtained in the questionnaire on height measurement, or dose and duration of iron and folic acid supplementation, or HIV-counseling and testing. The information was not checked with an ANC card/book if available.

Tetanus toxoid injections are given during pregnancy to prevent neonatal tetanus, a frequent cause of infant deaths when sterile procedures are not observed in cutting the umbilical cord following delivery. Women in their first pregnancy typically receive 2 doses (TT1–TT2) with 1 month in between; subsequent doses are recommended after a minimum of half a year (TT3) or one year apart (TT4–TT5), and are in general given during ANC visits for subsequent pregnancies [13,28]. Five doses are considered to provide life-long immunity. Among 570 women who could recall the number of tetanus doses received, 97% received at least one dose; 46% received only one dose, 43% received 2 doses, and 8% received ≥3 doses. Although any coverage with tetanus vaccination was generally high, among primiparae 29% reported one dose only, whereas 41% of para ≥ 5 reported ≥ 2 doses.

### Care during delivery

Most women (83%) delivered outside of a health facility. Of these, 80% delivered in their own house, 18% in the house of a TBA and 3% on their way to a health facility. The most frequent reason for not attending a health facility for delivery was lack of means of transport, in particular at night (49%). Other important barriers were fast progression of labor (47%), and expense (28%). Fourteen percent of women did not think facility attendance was necessary; reasons given for this included previous uneventful home delivery, preferred home deliveries, or had made arrangements with TBAs or another person to attend the delivery. A small subset (3%) reported anticipation of unpleasant treatment at a health facility as a reason not to attend. Of note, 64% of those who delivered outside a health facility were aware of the potential risks, and could identify one or more complications that could occur. Among women who did not visit an antenatal clinic, only 1 woman (1.6%) delivered in a health facility; this was 10.2% among the 266 women who made 1–3 visits, and 27.2% among the 305 women who made 4 or more visits (Trend test *P *< 0.001). In multivariate analysis, factors associated with delivery outside a health facility included: age ≥ 30 years, parity ≥ 5, low/medium SES, < 8 years of education, and > 1 hour walking distance from the hospital (Table 3).

**Table 3:** Association between maternal characteristics and a birth outside a health facility, Asembo/Gem, Western Kenya, December 2002*

	n (%*) who delivered in a health facility (N = 111)	n (%*) who delivered outside a health facility (N = 524)	Factors associated with birth outside a health facility, univariate analysis, OR (95% CI)	Factors associated with birth outside a health facility, multivariable analysis, AOR (95% CI)
Age				
< 18 years	10 (9.0)	30 (5.7)	0.78 (0.36–1.67)	0.77 (0.28–2.13)
18–19 years	19 (17.1)	61 (11.6)	0.83 (0.46–1.49)	0.90 (0.45–1.82)
20–29 years	64 (57.7)	247 (47.1)	Reference	Reference
30–34 years	10 (9.0)	98 (18.7)	**2.54 **(1.25–5.15)	1.65 (0.69–3.94)
> 34 years	8 (7.2)	88 (91.7)	**2.85 **(1.31–6.18)	1.25 (0.48–3.24)
Parity				
Para 1	30 (27.0)	81 (15.5)	0.80 (0.48–1.33)	0.98 (0.47–2.05)
Para 2–4	56 (50.5)	190 (36.3)	Reference	Reference
Para ≥ 5	25 (22.5)	253 (48.3)	**2.98 **(1.80–4.96)	1.63 (0.81–3.26)
Marital status				
Single/Widow	22 (19.8)	66 (12.6)	**0.58 **(0.34–0.99)	0.90 (0.45–1.81)
Married	89 (80.2)	457 (87.4)	Reference	Reference
Socio-economic status				
Low/medium SES	49 (46.7)	303 (58.8)	**1.63 **(1.07–2.49)	**1.61 **(1.02–2.54) †
High SES	56 (53.3)	212 (41.2)	Reference	Reference
Education level				
< 8 years	44 (41.5)	298 (58.4)	**1.98 **(1.30–3.03)	**1.68 **(1.04–2.69) †
≥ 8 years	62 (58.5)	212 (41.6)	Reference	Reference
Residence				
Asembo	60 (54.1)	259 (49.4)	0.83 (0.55–1.25)	
Gem	51 (45.9)	265 (50.6)	Reference	
Travel to ANC				
Walking < 1 hour	74 (66.7)	251 (47.9)	Reference	Reference
Walking 1 hour	18 (16.2)	126 (24.0)	**2.06 **(1.18–3.61)	1.71 (0.95–3.07)
Walking > 1 hour	10 (9.0)	117 (22.3)	**3.45 **(1.72–6.92)	**2.75 **(1.33–5.68)
Used bus or bicycle	9 (8.1)	30 (5.7)	0.98 (0.45–2.16)	1.24 (0.50–3.07)
Previous child-death				
≥ 3 times	3 (2.7)	44 (8.4)	**4.18 **(1.27–13.81)	1.35 (0.36–5.10)
1 or 2 times	23 (20.7)	182 (34.7)	**2.26 **(1.37–3.71)	1.60 (0.89–2.88)
None	85 (76.6)	298 (56.9)	Reference	Reference
Previous stillbirth				
Yes	14 (12.6)	59 (11.3)	0.88 (0.47–1.64)	
No	97 (87.4)	465 (88.7)	Reference	

Abbreviations: OR, odds ratio; AOR, adjusted odds ratio; CI, confidence interval; SES: socio-economic status. Significant odds ratios printed in bold.

*Column percentages

†The introduction of number of ANC visits (≥ 4 visits, 1–3 visits vs. no visit as reference) in the multivariate model reduced the AOR of Low/medium SES (AOR 1.36, 95% CI 0.85–2.17), and education level < 8 years (AOR 1.46, 95% CI 0.90–2.38), but did not affect the other variables in the model. The AOR for ≥ 4 visits was 15.08 (95% CI 1.99–114), and for 1–3 visits was 2.81 (1.68–4.69).

Among all women, only 17% were attended to by a professionally trained provider (doctor, nurse, midwife, or clinical officer) and TBAs assisted another 36% of women. A disturbingly high proportion of women (29%) were attended to by an untrained family member, friend or neighbor, and 18% of women delivered completely unattended (Figure 2). In multivariate analysis, women who delivered on their own were more likely to be of parity ≥ 5 (AOR 5.7, 95% CI 2.8–11.6), whereas women who had lost a child previously (any child death) were less likely to deliver on their own (AOR 0.5, 95% CI 0.3–0.9). Age, education level, SES, marital status, and distance to ANC were not significant factors in this model (data not shown).

**Figure 1:**
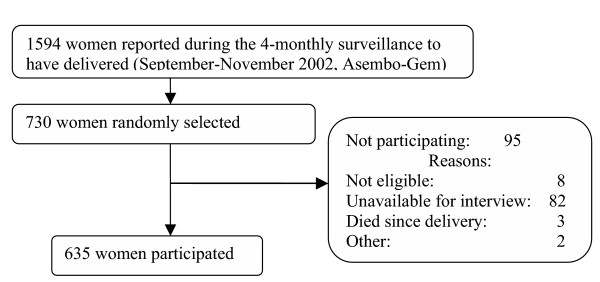
Flow chart of the survey population, Asembo/Gem, Western Kenya, December 2002.

## Discussion

In this survey in a rural area in western Kenya, 9 out of 10 women reported at least one ANC visit during their last pregnancy; however, two-thirds of these women began attending the ANC in the third trimester, and only half of these women made the recommended number of 4 visits. Services provided by the various ANCs were not optimal, with a low coverage of intermittent preventive treatment with sulfadoxine-pyrimethamine, and supplementation of iron and folic acid. The proportion of women assisted during delivery by a professionally trained provider (17%) was similar to the proportion of women who delivered on their own (18%). Untrained persons and TBAs assisted in the majority of deliveries (65%).

The high ANC attendance (90%) in this rural area is encouraging, and is similar to a report for the whole of Kenya (88%) [29]. Having more than 8 years of education and being of higher socio-economic status were the most important factors associated with ANC attendance; these findings are similar to those from an evaluation of antenatal care attendance in developing countries [13]. Although the perceived expense of the ANC may hinder attendance, it is uncertain that free antenatal care would increase coverage substantially because transport costs, physical inability to travel long distances, and a perceived poor quality of care would remain barriers. Unlike Nigeria and Uganda, where the husband played an important role in determining their partner's ANC attendance, in our study most women made an independent decision to attend [17,30].

There were fewer services offered by the ANCs attended by the women in this study compared to the whole of Nyanza Province; participants in this survey were half as likely to receive information on danger signs in pregnancy, or to receive a urine test (30% versus 14%, and 35% versus 16% respectively) than participants in the 2003 Demographic and Health Survey (DHS), and they were less likely to report a blood pressure measurement (67% versus 76%, *P *= 0.001) [29]. Women appreciated the information and advice received at the ANC. The main topics were care during pregnancy and care for the newborn. However, few women attended a health talk (14%), and other essential topics such as place of delivery, making an individual birth plan, family planning, malaria, and HIV/AIDS prevention received little attention. Information on the effect of health education on maternal and perinatal outcome in sub-Saharan Africa is limited; a study in Tanzania assessing the effect of group and individual counseling on the prevention of anemia did not see an additional effect of this approach above the intervention of ensuring an adequate supply of hematinics and malaria prophylaxis [31]. Other services with proven benefit had a low coverage in this survey as well; barely half of the women received hematinic supplements, one out of 5 women received one or more doses of sulfadoxine-pyrimethamine for malaria, and an antihelmintic treatment was received by 3%. This is in stark contrast with the needs as confirmed during a cross-sectional survey in July 2003 among 673 pregnant women in the same area; 36% of the women were malaria parasitemic (52% among primigravidae), 53% were anemic (hemoglobin < 11 g/dl) and 76% of the 391 women who had brought a stool sample were infected with a nematode (van Eijk *et al*., unpublished). In our study, we did not investigate the reasons why many ANC visits passed without provision of these services, e.g. if there was a lack of supply of drugs; this is a focus of future evaluations.

Consistent with a report from a neighboring area, only 37% of the woman started attending ANC in the first or second trimester [32]. Low ANC attendance in the first trimesters has also been reported in other African and developing countries [19,29,30,33,34]. Late ANC attendance may preclude women from benefiting fully from preventive strategies, such as iron and folic acid supplementation, treatment of helminthic infections, and intermittent preventive treatment with sulfadoxine-pyrimethamine for malaria in pregnancy. Late attendance was associated with a perceived lack of services at the ANC. As summarized in table 2, provision of services in this area was generally poor and worse than that reported in the national ANC service profile in the most recent DHS [29]. To encourage earlier ANC attendance, service delivery must be improved.

Despite the fact that 90% of the women reported attending antenatal care, fewer than 2 in 10 gave birth in a health facility. This is lower than the national estimate of 40% for Kenya and 39% for Nyanza Province [29], although higher than that reported in some other African countries [14]. Observed contributing factors for a home delivery included the fast progression of labor, distance, difficulty of (night) travel, and cost. Distance was a barrier for facility delivery but not for ANC attendance. This may be because not every ANC facility has 24-hour maternity service, so the distance to reach a health facility with maternity service may be greater than for antenatal care. In addition, given the urgent nature of deliveries, there may be less time to cover the distance. Other work has confirmed the importance of distance on access to maternity care [18,34].

We were surprised at the high proportion of women who gave birth without any assistance. Our estimate of 18% was nearly twice that observed in the most recent Kenya DHS (National estimate 8.0%, Nyanza Province 9.8%) and in a study in Mbeere District, Kenya (6.5%) [19,29]. Unassisted delivery was particularly common among grande multiparae, a high-risk group in obstetrics [19,29]. The lack of any attendant makes it difficult to seek assistance in the event of life-threatening complications. Women should be strongly encouraged to deliver with assistance. The promotion of a delivery plan may be a good step towards sensitizing women on this issue.

TBAs frequently form the backbone of maternity services in rural areas, and in our survey they attended about one-third of deliveries. It has been suggested that training of TBAs could reduce maternal and perinatal mortality, but recent data have not supported this strategy [20,35,36]. Some participants preferred TBAs because of greater flexibility in payment.

Limitations of this survey include the fact that the sample of women who could not be found were younger than those enrolled, and young women were more likely to visit the ANC; however, even if all excluded women would have visited the ANC, the estimate of the prevalence of having attended ANC at least once would not have changed substantially. We did not cross check the information provided by the women during the interviews with ANC card data; thus, services actually provided could have been underreported. Lastly, only women who survived their last delivery were able to participate in this survey.

## Conclusion

The Safe Motherhood Initiative promotes antenatal care, and skilled assistance (defined as a midwife, nurse trained as midwife, or a doctor) during childbirth [37,38]. Based on this definition, there is much to be done to achieve 'Safe Motherhood' in rural areas of western Kenya: a trained provider attended fewer than 1 in 5 deliveries. The discrepancy between the national and provincial estimates of unassisted deliveries or deliveries without skilled assistance and our findings illustrate that these estimates may underestimate the situation in the rural areas. Safe motherhood strategies promote counseling of clients on danger signs and the development of an individual birth plan; pregnant women and their relatives are encouraged to arrange transport, money and a companion before onset of labor [28]. This survey indicates that the situation in western Kenya needs urgent improvement. The Kenya Ministry of Health is working on expanding focused antenatal care and prevention of malaria in pregnancy in rural areas [28]. This survey can provide baseline data for this rural area against which efforts to improve services in the ANCs can be evaluated.

## Competing interests

The author(s) declare that they have no competing interests.

## Authors' contributions

Amve was involved in the design of the study, in analysis and interpretation of the data, and in drafting the paper. HB and IEB were involved in the analysis and interpretation of the data and in drafting the paper. FO, JGA and KA were involved in data collection, and revision of the manuscript. DHR was involved in statistical analysis and interpretation of the data, and revising the manuscript. LS was involved in design, interpretation of the data and review of the manuscript. KAL was involved in conception and design, interpretation of the data and drafting and reviewing the manuscript. Each author has given final approval of the version to be published.

**Figure 2:**
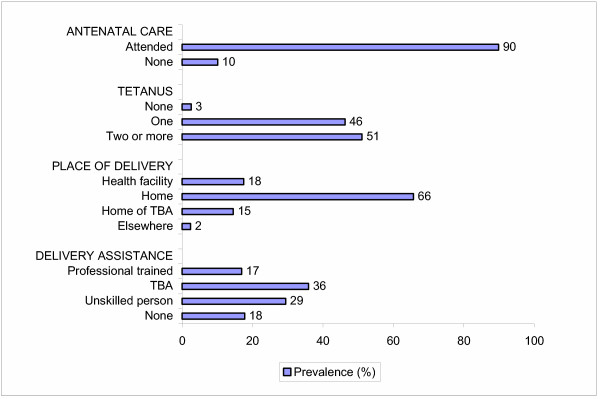
ANC attendance, number of tetanus doses, place of delivery and attendants, Asembo/Gem, Western Kenya, December 2002.
